# Longitudinal analysis of 5-year refractive changes in a large Japanese population

**DOI:** 10.1038/s41598-022-06898-x

**Published:** 2022-02-21

**Authors:** Masaki Takeuchi, Akira Meguro, Masao Yoshida, Takahiro Yamane, Keisuke Yatsu, Eiichi Okada, Nobuhisa Mizuki

**Affiliations:** 1 Department of Ophthalmology and Visual Science, Yokohama City University Graduate School of Medicine, Yokohama, Kanagawa 236-0004 Japan; 2 Department of Advanced Medicine for Ocular Diseases, Yokohama City University Graduate School of Medicine, Yokohama, Kanagawa 236-0004 Japan; 3 Department of Public Health, Kyorin University School of Medicine, Mitaka, Tokyo 181-8611 Japan; 4 Department of Medical Science and Cardiorenal Medicine, Yokohama City University School of Medicine, Yokohama, Kanagawa 236-0004 Japan; 5 Minna no Naika Clinic Ningyocho-Suitengu, Chuo-ku, Tokyo 103-0013 Japan; 6 Okada Eye Clinic, Yokohama, Kanagawa 234-0054 Japan

**Keywords:** Refractive errors, Epidemiology

## Abstract

Refractive changes are reportedly affected by age, sex, and current refractive error. To clarify the pattern of refractive changes in a Japanese population, we conducted a 5-year follow-up longitudinal analysis of spherical equivalent (SE) refractive changes with stratification by sex, age, and SE in 593,273 eyes from Japanese individuals ages 3–91 years. The 5-year SE change with myopic shift dramatically increased over time after age 4 years, and the largest change was observed in both males and females who were age 8 years at baseline [males: − 2.654 ± 0.048 diopters (D); females: − 3.110 ± 0.038 D]. During school age, the 5-year myopic change was greater in females than in males, and emmetropic and low-to-moderate myopic eyes underwent larger myopic changes than hyperopic and high-to-severe myopic eyes. After the peak at age 8 years, the 5-year myopic change gradually declined with age and fell below − 0.25 D at age 27 in males and age 26 years in females. The 5-year SE changes transitioned from a myopic to a hyperopic shift at age 51 in both sexes, and hyperopization advanced more quickly in hyperopic eyes. Our findings highlight the importance of myopia prevention in school-aged children.

## Introduction

Myopia, which is commonly defined as a spherical equivalent (SE) refraction of ≤ − 0.50 diopters (D), is the most common refractive error of the human eye in the modern world and has a significant impact on public health^1^. High myopia is associated with a higher risk of various ocular diseases, including retinal detachment, glaucoma, cataracts, and macular degeneration, that can lead to visual impairment or blindness^2^. The prevalence of myopia is generally high, particularly in Asian countries^3–7^, and has dramatically increased worldwide in the last several decades^8^. By the year 2050, myopia and high myopia are respectively predicted to affect approximately 50% and 10% of the world population (almost 5 billion and 1 billion people)^8^. Thus, it is important to clarify the pattern of refractive error changes in general populations.

Refractive errors change with postnatal growth^9–11^. Although neonates are generally hyperopic, refractive errors usually become low to mild hyperopic, emmetropic, or low myopic by the time children enter elementary school^12,13^. Myopic changes then progress, with a decrease in the number of children with hyperopia and an increase in those with myopia^12,14^. Myopia progression becomes noticeable at ages 10–14 years and continues a mild course until it ceases shortly after age ~ 22–23 years^15,16^. According to the Beaver Dam Eye Study in a population of U.S. adults, prevalence rates of myopia and hyperopia are respectively 43.0% and 22.1% in those ages 43–45 years and 14.4% and 68.5 in those age ≥ 75 years^17^. Similar trends have been reported in population-based studies with other U.S. and Australian adult community residents, including in the Framingham Eye Study^18^, Baltimore Eye Study Survey^19^, and Blue Mountains Eye Study^20^, all suggesting that myopia is common in adults and hyperopia becomes more common with age. Studies in adult Japanese populations have shown similar patterns^21,22^. These studies were cross-sectional surveys in community residents and provided little information about changes over time and included limited control for possible confounders.

Longitudinal studies are important for tracking refractive progression in detail and clarifying the relationship between progression and possible confounders. Mäntyjärvi et al. followed up 72 boys and 107 girls ages 7–15 years for 5–8 years in a Finnish population and found mean annual changes in refractive errors of − 0.12 D in hyperopic and − 0.55 D in myopic eyes, suggesting that children with myopia experience faster myopic progression than children with hyperopia^15^. In 124 Danish school children ages 9–12 years, Jensen followed up SE refractive errors for 8 years and found that the earlier myopia started, the more it progressed^23^. Xiang et al. conducted a 4-year follow-up analysis of refraction tests in 607 Chinese twins ages 7–15 years and reported that the annual SE refractive changes in younger children (ages 7–11 years) were larger than in older children (ages 12–15 years)^24^. In studies of SE refractive changes over 2 years in 2053 new medical students in China^25^, with a mean age of 18.27 ± 1.83 years, and in 166 clinical microscopy technicians in Britain, ages 21–55 years^26^, the higher the myopic level at study start, the faster the myopic shift. Few previous studies have investigated SE refractive changes in a Japanese population. In one study evaluating the effectiveness of progressive addition lenses and single vision lenses on myopia progression over 3 years in 86 Japanese myopic children ages 6–12 years, greater myopia progression was observed in the more myopic eyes and at younger ages^27^. These longitudinal studies have investigated refractive changes over time, but the sample sizes did not allow for sufficient statistical power to fully elucidate the pattern of changes. Moreover, because myopia progression at different life stages depends on age and current refractive error, refractive changes should be assessed in a large cohort finely stratified by age, sex, and refractive error, but no such reports have been published.

We conducted the largest longitudinal study of SE refractive changes in a Japanese cohort to date, stratified by age, sex, and SE. The findings from this longitudinal study covering a wide range of ages will aid understanding of SE refractive changes throughout the lifetime for each baseline SE.

## Results

### Five-year refractive changes

**Figure 1:**
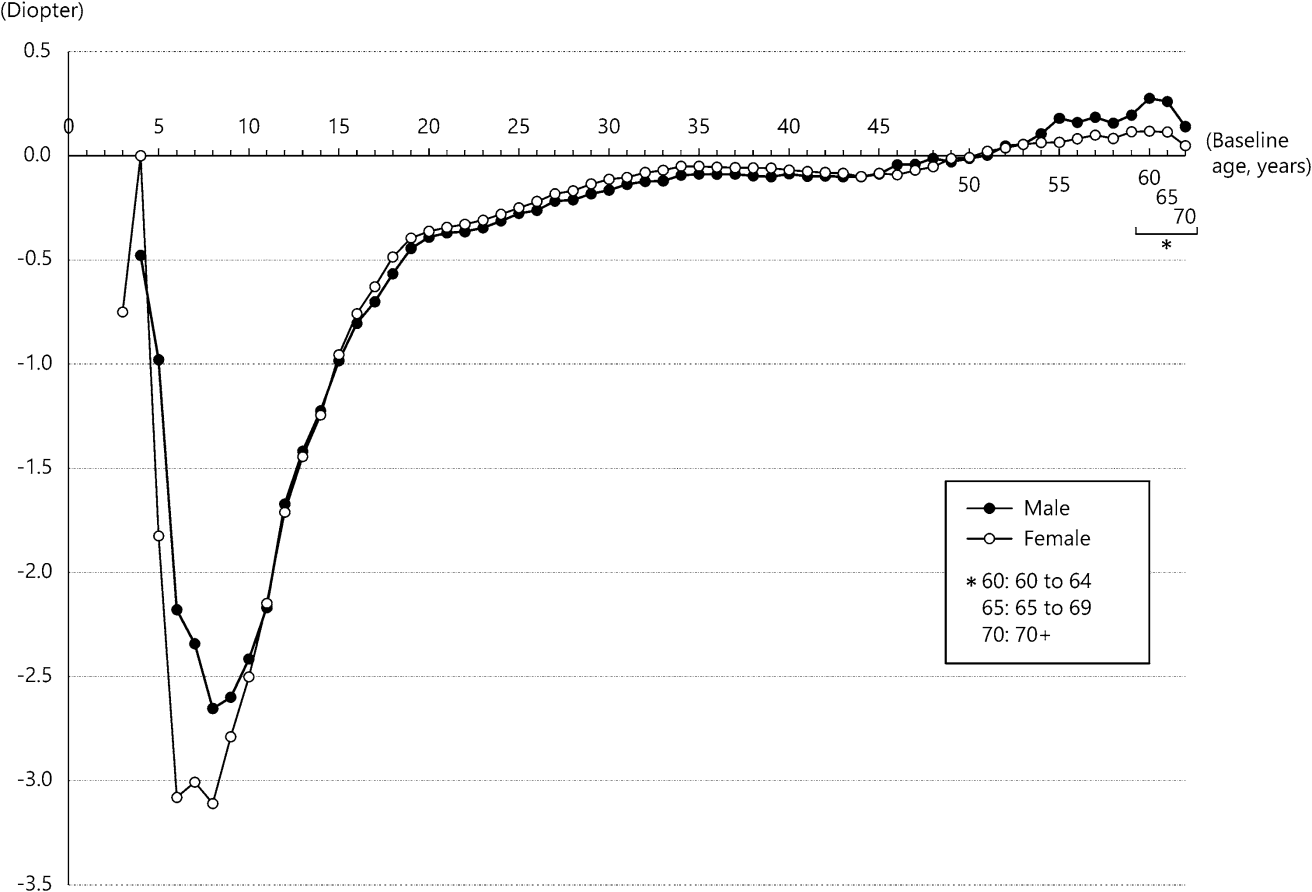
5-year mean change in spherical equivalent (SE) refractive error for each age at baseline. The x-axis represents ages at baseline, in years. The y-axis represents the magnitude of 5-year mean SE refractive changes at each baseline age in males (black circles) and females (white circles).

We retrospectively followed 593,273 eyes from Japanese individuals ages 3–91 years for 5 years (Supplementary Table S1). Of these eyes, 228,074 (38.4%) were from males and 365,199 (61.6%) were from females. We investigated 5-year SE refractive changes across age in years and by sex (Fig. 1 and Supplementary Table S2).

In females at age 3 years, the mean change (± standard error) in SE in the following 5 years was − 0.750 ± 0.395 D, although this myopic change did not reach significance because of the small sample size. For those age 4 years at baseline, the 5-year mean SE change in males was − 0.479 ± 0.162 D, whereas females showed no mean change in SE (0.000 ± 0.279 D). Afterward, the 5-year mean SE change with myopic shift dramatically increased with age, exceeding − 2.00 D at the age of 6 years and peaking at 8 years in both males and females. The largest 5-year SE mean change was observed in both males and females who were age 8 years at baseline (males: − 2.654 ± 0.048 D; females: − 3.110 ± 0.038 D). After the peak at age 8 years at baseline, the 5-year mean SE change began to decrease, and 5-year mean SE changes exceeding − 2.00 D up to age 11 years and − 1.00 D up to age 14 years were observed in both males and females.

After the late teens, the rate of the 5-year mean SE change with myopic shift decreased slowly with increasing age, falling below − 0.50 D at age 19 years in males and age 18 years in females. It declined to − 0.25 D, the minimum value for correction with eyeglasses, at age 27 years in males and age 26 years in females, and to − 0.10 D at age 34 years in males and age 32 years in females. At age 51 years, the 5-year mean SE change transitioned from a negative to a positive value in both males and females, indicating a shift from myopia to hyperopia. After that, the 5-year mean SE change with a hyperopic shift tended to slowly increase with age up to around the late sixties in both males and females. Among those ages 51 to 71 years, the largest 5-year SE mean change with a hyperopic shift was observed at 64 years in males (+ 0.394 ± 0.040 D) and 67 years in females (+ 0.202 ± 0.039 D). From the age of 72 years, the direction of the 5-year mean SE change occasionally showed a myopic shift. The statistical analysis showed that SE significantly changed from baseline age to the 5-year follow-up in males ages 4–27 years and females ages 5–27 years (*P* < 0.05 after Bonferroni correction) (Supplementary Table S2).

When comparing the rate of the 5-year mean SE change between males and females, myopic shift was greater in females at ages 5 to 14 years (except for age 11 years), which was associated with a large 5-year mean SE change, especially in females compared with males from ages 5 to 8 years, with significant sex differences in 5-year myopic change at ages 7 and 8 years. In contrast, for those ages 15 to 45 years, the 5-year myopic change was slightly greater in males than in females, and sex differences in the 5-year myopic change for ages 15 to 39 and at age 41 were significant. At ages 54 to 79 years, the 5-year hyperopic change was greater in males than in females, except at ages 67, 73, 75, and 76 years, and sex differences in the 5-year hyperopic change were significant at most ages (Supplementary Table S2).

### Five-year refractive changes stratified by age group and refractive error level

**Figure 2:**
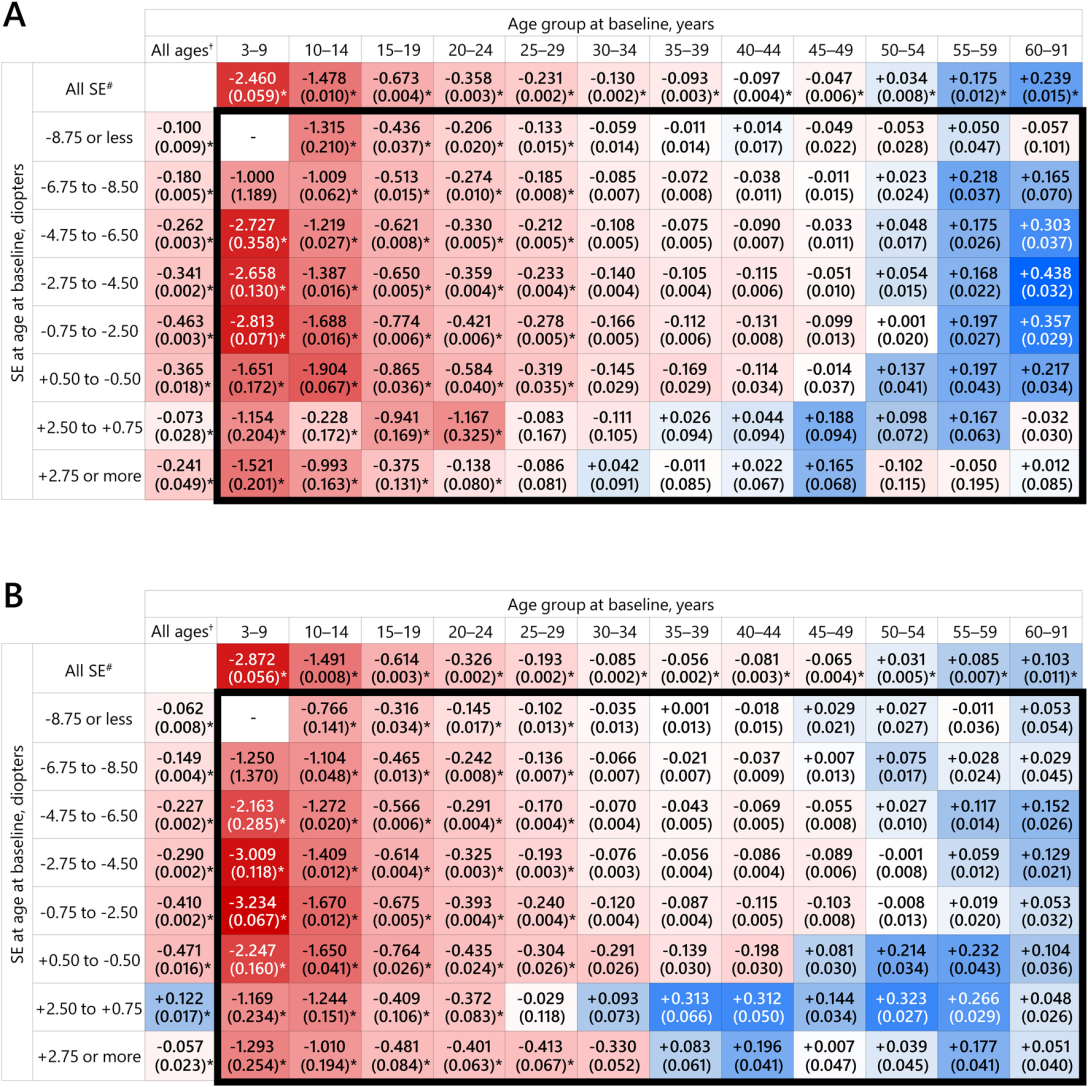
5-year mean change in spherical equivalent (SE) refractive error stratified by age group and SE level in males (**A**) and females (**B**). The 5-year mean SE change value and standard error (in parentheses) in each category classified by age group and SE level are shown within the respective square. The categories stratified by both age group and SE level are surrounded by a thick black line. Larger 5-year myopic and hyperopic changes are indicated by brighter red and blue, respectively. ^†^5-year mean SE change (standard error) adjusted by analysis of covariance (ANCOVA) with baseline age as an independent variable. ^#^5-year mean SE change (standard error) adjusted by ANCOVA with baseline SE as an independent variable. ^*^5-year mean SE changes significant at *P* < 0.05 after Bonferroni correction based on the number of categories stratified by age and SE groups.

Figure 2 shows 5-year mean SE refractive changes stratified by age group and SE level at baseline age. When the participants were stratified by SE levels, the largest 5-year mean SE change in males was observed at SE − 0.75 to − 2.50 D (− 0.463 ± 0.003 D), followed by + 0.50 to − 0.50 D, − 2.75 to − 4.50 D, and − 4.75 to − 6.50 D. The largest 5-year mean SE change in females was observed at SE + 0.50 to − 0.50 D (− 0.471 ± 0.016 D), followed by − 0.75 to − 2.50 D, − 2.75 to − 4.50 D, and − 4.75 to − 6.50 D. In contrast, the smallest 5-year myopic change in males was observed at SE + 2.50 to + 0.75 D (− 0.073 ± 0.028 D), followed by − 8.75 D or less, and − 6.75 to − 8.50 D. In females, 5-year hyperopic change was observed at SE + 2.50 to + 0.75 D (+ 0.122 ± 0.017 D) and the smallest 5-year myopic change was observed at + 2.50 D or more (− 0.057 ± 0.023 D), followed by − 8.75 D or less, and − 6.75 to − 8.50 D.

When the participants were stratified by age group, in both males and females, 5-year mean SE change showed a myopic shift from ages 3–9 years to 45–49 years, with the largest 5-year myopic change at ages 3–9 years (males: − 2.460 ± 0.059 D; females: − 2.872 ± 0.056 D), followed by ages 10–14, 15–19, 20–24, 25–29, and 30–34 years, in order. The 5-year mean SE change fell below − 0.25 D, the minimum value for correction with eyeglasses, during ages 25–29 years in both males and females. Likewise in both males and females, from ages 50–54 to 60–91 years, the 5-year mean SE change showed a hyperopic shift, with those ages 60–91 years having the largest 5-year hyperopic change (males: + 0.239 ± 0.015 D; females: + 0.103 ± 0.011 D), followed by ages 55–59 and 50–54 years.

For those ages 3–9 years, the 5-year mean SE change exceeded − 2.00 D in SE levels from − 0.75 to − 6.50 D in males and from + 0.50 to − 6.50 D in females. The largest myopic change was observed at SE − 0.75 to − 2.50 D in both males and females (males: − 2.813 ± 0.071 D; females: − 3.234 ± 0.067 D), followed by − 4.75 to − 6.50 D and − 2.75 to − 4.50 D in males and − 2.75 to − 4.50 D, + 0.50 to − 0.50 D, and − 4.75 to − 6.50 D in females. In contrast, the smallest myopic change in males was observed at SE − 6.75 to − 8.50 D (− 1.000 ± 1.189 D), followed by + 2.50 to + 0.75 D and + 2.75 D or more, and at + 2.50 to + 0.75 D (− 1.169 ± 0.234 D) in females, followed by − 6.75 to − 8.50 D and + 2.75 D or more. Of these, myopic changes in SE groups from “ + 2.75 D or more” to “− 4.75 to − 6.50 D” were significant in both males and females.

In the 10–14-year age group, the largest 5-year myopic change in males was observed at SE + 0.50 to − 0.50 D (− 1.904 ± 0.067 D), followed by − 0.75 to − 2.50 D and − 2.75 to − 4.50 D, and at − 0.75 to− .50 D (− 1.670 ± 0.012 D) in females, followed by + 0.50 to − 0.50 D and − 2.75 to − 4.50 D. The smallest 5-year myopic change in males was observed at SE + 2.50 to + 0.75 D (− 0.228 ± 0.172 D), followed by + 2.75 D or more and − 6.75 to − 8.50 D, and at − 8.75 D or less (− 0.766 ± 0.141 D) in females, followed by + 2.75 D or more and − 6.75 to − 8.50 D. In those ages 10–14 years, myopic changes in all SE groups were significant in both sexes.

In those ages 15–19 years, the largest 5-year myopic change in males was observed at SE + 2.50 to + 0.75 D (− 0.941 ± 0.169 D), followed by + 0.50 to − 0.50 D and − 0.75 to − 2.50 D, and the smallest change was at SE + 2.75 D or more (− 0.375 ± 0.131 D), followed by − 8.75 D or less and − 6.75 to − 8.50 D. In females, the largest myopic change was observed at SE + 0.50 to − 0.50 D (− 0.764 ± 0.026 D), followed by − 0.75 to − 2.50 D and − 2.75 to − 4.50 D, and the smallest change was at SE − 8.75 D or less (− 0.316 ± 0.034 D), followed by + 2.50 to + 0.75 D and − 6.75 to − 8.50 D. The age group 20–24 years also showed the largest myopic change at SE + 2.50 to + 0.75 D in males (− 1.167 ± 0.325 D) and + 0.50 to − 0.50 D (− 0.435 ± 0.024 D) in females and the smallest change at + 2.75 D or more in males (− 0.138 ± 0.080 D) and − 8.75 D or less in females (− 0.145 ± 0.017 D). In the 15–19- and 20–24-year age groups, myopic changes in all SE groups were significant in both sexes. Additionally, the age group 25–29 years had significant myopic changes in all SE groups except for + 2.50 to + 0.75 D and + 2.75 D or more in males and + 2.50 to + 0.75 D in females.

For males ages 30–34 years and females ages 35–39 years, the 5-year mean SE change declined below − 0.25 D at all SE levels, and no significant myopic change was observed in any SE groups for ages 30–34 years and older in either sex. A 5-year hyperopic change was observed among the age groups from 30–34 years to 60–91 years in both males and females. In males, the largest hyperopic change was observed at SE + 2.75 D or more for ages 30–34 years, + 2.50 to + 0.75 D for ages 35–39 to 45–49 years, + 0.50 to − 0.50 D for ages 50–54 years, − 6.75 to − 8.50 D for ages 55–59 years, and − 2.75 to − 4.50 D for ages 60–91 years. For females, the largest hyperopic change was observed at SE + 2.50 to + 0.75 D at ages 30–34 to 55–59 years, and − 4.75 to − 6.50 D at ages 60–91 years. However, these hyperopic changes did not reach statistical significance because of the relatively small sample sizes of the adult groups showing the hyperopic change.

## Discussion

To investigate patterns of SE refractive changes in a Japanese population, we conducted this large-scale population-based study and analysed longitudinal data for 593,273 eyes from individuals whose SE refractive errors were followed up for 5 years. Because age and current refractive error affect myopia progression at different life stages, we stratified the data by age and refractive error.

The results show that school-aged children underwent a large myopic change. The 5-year mean SE change exceeded − 2.00 D at the baseline age range of 6 to 11 years in both males and females. The largest 5-year change was observed in those who were age 8 years at baseline (males: − 2.654 ± 0.048 D; females: − 3.110 ± 0.038 D). In addition, stratification by age groups showed that those ages 3–9 years had the largest 5-year myopic change (males: − 2.460 ± 0.059 D; females: − 2.872 ± 0.056 D), followed by those ages 10–14 years (males: − 1.478 ± 0.010 D; females: − 1.491 ± 0.008 D). A previous study with Chinese children ages 6–15 years identified 5-year mean SE refractive changes of − 2.21 ± 1.87 D and showed that myopic progression was associated with younger age^28^. Another study in India of people ages 1–30 years showed an age effect on myopia progression and identified the largest annual change in SE in children ages 6–10 years, followed by those ages 0–5 and 11–15 years^29^. In a longitudinal multi-ethnic study in children ages 7 to 13 years, the peak progression in the first year of myopia onset was − 0.58 D at age 7 years, with decreasing annual SE refractive change as the myopia onset age increased^30^. Thus, our findings in school-aged children of the Japanese population are similar to previous results in other countries.

We also found that myopic progression in Japanese children ages 5 to 14 years was faster in females than in males, with much larger sex-based differences in those who were ages 5 to 8 years at baseline. We observed 5-year mean SE changes exceeding − 3.00 D only in girls ages 6, 7, and 8 years but not in boys. Previous studies also have shown a significantly faster myopic progression in school-aged females compared to males in China, Singapore, and the United States^28,30–33^, but studies from Australia and the UK have shown only slightly faster progression in female than in male school-aged children^34,35^. Based on the current and previous findings, we confirmed that females have a faster myopic shift than males during school age. This faster myopic shift could result from average lifestyle differences between males and females, with girls spending more time on close-up visual work such as reading and doing homework^36–38^, which are thought to be risk factors for myopia^39,40^, and boys spending more time on outdoor activities, which are thought to be protective factors against myopia^40,41^. In addition, differences between males and females in sex hormone levels and physical structural changes in height and weight may also underlie sex differences in SE refractive changes^42^.

Our stratification analysis of data for the Japanese school-aged children by baseline SE levels revealed that the emmetropic, low myopic, mild myopic, and moderate myopic groups with a baseline SE ranging from + 0.50 to − 6.50 D had a larger 5-year myopic change than the hyperopic, high myopic, and severe myopic groups with a baseline SE of + 0.75 D or more or − 6.75 D or less. Many previous studies have reported a relationship between myopia progression and baseline SE in school-aged children (Supplementary Table S3). For instance, Fan et al. showed that a higher degree of myopia at baseline was related to steeper annual myopic progression in children ages 5–16 years in Hong Kong^3^. Wong et al. reported a faster annual myopic progression with moderate myopia (− 6.0 D < SE ≤ − 3.0 D) compared to mild myopia (− 3.0 D < SE ≤ − 0.5 D) in children age < 17 years in the UK^35^. Additionally, Verkicharla et al., in their study in India, found the greatest annual myopic progression with severe myopia (SE < − 9.0 D), followed by high (− 9.0 D ≤ SE < − 6.0 D), moderate (− 6.0 D ≤ SE < − 3.0 D), and mild myopia (− 3.0 D ≤ SE ≤ − 0.5 D), in children age ≤ 15 years^29^. Furthermore, Zhao et al. reported that myopic progression was associated with higher myopic or hyperopic refractive error at baseline in a 28.5-month follow-up of Chinese children ages 5–13 years^32^. In a study with a 5- to 6-year follow-up of two cohorts of younger and older Australian children (median ages 6.7 ± 0.4 and 12.7 ± 0.4 years, respectively), French et al. reported that children with ametropia (myopia or hyperopia) at baseline had steeper myopic progression compared to children with emmetropia and that myopic shift with myopia was greater than with hyperopia^34^. Saw et al., in an analysis of data from a 3-year follow-up of children in Singapore, found the fastest myopia progression with higher myopia (− 6.0 D < SE ≤ − 3.0 D), followed by low myopia (− 3.0 D < SE ≤ − 0.5 D), high myopia (SE ≤ − 6.0 D), and emmetropia (SE > − 0.5 D)^31^. Based on these findings, baseline SE certainly affects myopia progression in school-aged children, but the reported degree of this effect is not necessarily the same among the current and previous studies. One reason for these divergences may be differences in age ranges and genetic, environmental, and lifestyle backgrounds among the cohorts. Considering the importance of myopia prevention in school-aged children, further detailed study is required to clarify the relationship between baseline SE and myopia progression.

In the current study, the 5-year myopic change peaked in childhood and decreased with age regardless of baseline SE values. Because the minimum value for eyeglass correction is generally 0.25 D, a 5-year SE change falling below − 0.25 D was defined as cessation of clinical myopic changes. We found that the 5-year myopic change was slightly larger in males than in females at ages 15 to 45 years and that clinical myopic changes stopped at age 27 years in males (− 0.217 ± 0.006 D) and age 26 years in females (− 0.220 ± 0.005 D). These findings indicate that clinical myopic changes cease earlier in females than in males in a Japanese population. When we stratified the participants by SE levels, however, the 5-year mean SE change fell below − 0.25 D at all SE levels after ages 30–34 years in males and 35–39 years in females, demonstrating that baseline SE level affects myopic change even in adults and suggesting a difference by baseline SE in the age at which myopia progression stops.

The 5-year mean SE changes transitioned from a myopic to a hyperopic shift at age 51 years in both males and females in our Japanese population. Among the SE groups, hyperopization for both males and females in the + 2.50 to + 0.75 D and + 2.75 D or more groups was faster than in other SE groups, and the 5-year SE changes in these groups became hyperopic at ages 30–39 years. These findings indicate that hyperopization progresses with age and advances more quickly in hyperopic eyes. These hyperopization trends can be attributed to latent hyperopia in hyperopic eyes that gradually becomes manifest over time because of an age-related decrease in accommodation^43^. The 5-year SE change remained in hyperopic shift to age 71 years, but at or after age 72 years, the changes varied with age, and some eyes showed a slight myopic shift in our Japanese population. The hyperopic shift after middle age and the myopic shift in old age have been reported previously in several ethnic groups (Supplemental Table S3). A 5-year follow-up study of SE changes in an elderly Chinese population showed 5-year mean SE changes of − 0.21 D, + 0.14 D, + 0.40 D, + 0.08 D, and − 0.34 D for those ages 30–39, 40–49, 50–59, 60–69, and 70 years or older, respectively^44^. A 10-year follow-up study of U.S. adults showed that younger people tended to shift toward being hyperopic, whereas older people shifted toward being myopic (mean SE change: + 0.48 D, + 0.03 D, and − 0.19 D for those ages 43–59, 60–69, and 70 years or older, respectively)^45^. In addition, a 9-year follow-up study of adults of African ancestry revealed a hyperopic shift in those ages 40–49 years at baseline (mean SE change: + 0.47 D) and a myopic shift in those ages 50 years or older (mean SE change: − 0.14 D, − 0.90 D, and − 0.78 D for ages 50–59, 60–69, and 70 years or older, respectively)^46^. Regarding the mechanism of SE changes after middle age, an aging-related decrease in lens refractive power has been suggested as a factor in hyperopia after middle age^44^. In contrast, the myopic shift in older age is thought to be caused by progression of nuclear cataracts and changes in ocular structure^47–49^. In the present study, SE changes after age 72 years varied with individual ages. Considering the effects of cataracts and changes in ocular structure on SE^47–49^, this difference in tendency to SE change at older ages may have resulted from individual differences in ocular conditions in our elderly population, and not solely because of small numbers in the older age group.

A potential weakness of the current study is that the participants were all ophthalmic patients who visited one eye clinic in Yokohama. Our findings may not be generalizable to the Japanese population as a whole because the distribution of age or illness in our patients may not reflect those of the general population. However, compared with findings from a recent epidemiological study of myopia prevalence in elementary and junior high school students in Tokyo^50^, the distribution of SE was similar among schoolchildren in the current study, indicating that our study cohort could reflect the general population in urban Japan.

Another weakness is that cycloplegics were not used in all participants but only for children whom we suspected to have refractive hyperopia or amblyopia. Cycloplegic autorefraction is important for obtaining accurate SE in all children to avoid the influence of active accommodation. This weakness is the most significant limitation of our study. Furthermore, we did not consider as covariates in these analyses a family history of refractive errors or environmental, lifestyle, or genetic factors, which all carry possible risk for refractive changes. In addition, the current study did not include ocular biometric parameters such as axial length, corneal curvature radius, and anterior chamber depth for the evaluation of SE change, which are reportedly related to the progression of myopia or hyperopia^51,52^. Although the large sample size (more than 500,000 eyes) is likely to reduce the effect of these limitations on public health statistical analyses, to overcome these limitations and understand refractive changes in detail, a population-based multicentre longitudinal study is needed that includes consideration of various risk and correlation factors for refractive changes in individuals universally receiving cycloplegics.

In conclusion, in this study in the Japanese population, the largest myopic change occurred at age 8 years in males and females, with a steeper change in females. Clinical myopization ceased at age 27 years in males and age 26 years in females. SE refractive changes transitioned from a myopic to a hyperopic shift at age 51 years in both males and females. Our findings can provide useful information for prediction of future SE change, and we have constructed an algorithm for predicting SE refractive changes after 5 years using baseline SE, baseline age, and sex as variables based on the data obtained from 593,273 eyes in the current study (manuscript in preparation). The prediction of future SE changes can guide uptake of preventive behaviours to prevent myopia progression, such as less time spent on work activities, especially in school-aged children. We expect our data and algorithm to contribute to prevention of myopia worsening.

## Methods

### Participants

We retrospectively investigated 5-year SE refractive changes in 593,273 eyes of patients ages 3–91 years who visited Okada Eye Clinic (Yokohama, Japan) for refractive error or suspected refractive error between January 2000 and December 2012. All participants were of Japanese ethnicity and living in Yokohama and its vicinity. Those with corneal diseases, a history of eye surgery, or with SE changes greater than ± 5.0 D within a year were excluded because of possible interference with refraction. Those with a history of any treatment with low-dose atropine eye drops, orthokeratology lens, or bifocal or progressive addition lens for prevention of myopia progression were also excluded. In terms of age stratification, patients were divided into groups at an interval of 5 years, except for a group of patients ≤ 9 years and those age ≥ 60 years (Supplementary Table S1). An Auto Refractometer (KR-3000; TOPCON, Tokyo, Japan) was used to measure SE refractive errors. Patients were divided into eight SE groups: + 2.75 D or more, + 2.50 D to + 0.75 D, + 0.50 D to − 0.50 D, − 0.75 D to − 2.50 D, − 2.75 D to − 4.50 D, − 4.75 D to − 6.50 D, − 6.75 D to − 8.50 D, and − 8.75 D or less (Supplementary Table S1). This study followed the tenets of the Declaration of Helsinki and was approved by the Ethics Committee of Okada Eye Clinic Institute. The Ethics Committee waived the need for informed consent due to the retrospective, observational, and anonymous nature of this study, according to the Ethical Guideline for Clinical Research issued by the Ministry of Health, Labour and Welfare, Japan.

### Statistical analysis

We investigated changes in SE refraction from baseline age to 5-year follow-up in each patient and analysed the 5-year mean SE change in each category classified by sex, baseline age, and/or baseline SE level using analysis of covariance (ANCOVA)^53^. When 5-year mean SE changes in each baseline age were analysed, the adjusted mean levels of 5-year SE changes were calculated from ANCOVA, with baseline SE as an independent variable. Similarly, when we analysed 5-year mean SE changes stratified by baseline age groups and baseline SE levels, we calculated the adjusted mean levels of 5-year SE changes using ANCOVA with baseline age and baseline SE as independent variables. *P* values to assess the significance of 5-year mean SE changes were calculated using ANCOVA, with Bonferroni correction for multiple testing and a corrected *P* < 0.05 was considered significant. The comparison of 5-year mean SE change between males and females was done using Welch’s t test. We excluded the categories with fewer than 10 participants from the statistical analysis. Age-standardized prevalences in SE groups were calculated using the direct method^54^, based on a 1985 Japanese model population^55^. All analyses were conducted using the SAS statistical software package (SAS Institute Inc., Cary, NC, USA).

## Supplementary Information

Supplementary Information.
