# Supplementary material for: Longitudinal analysis of 5-year refractive changes in a large Japanese population
Source: Sci Rep. 2022 Feb 21;12:2879. doi: 10.1038/s41598-022-06898-x (PMC8861094; doi:10.1038/s41598-022-06898-x)
Supplement: Supplementary file 1 — Supplementary Information. [file 41598_2022_6898_MOESM1_ESM.pdf]

SUPPLEMENTARY INFORMATION

Longitudinal analysis of 5-year refractive changes in a large Japanese population

Masaki Takeuchi[1,2], Akira Meguro[1,2*], Masao Yoshida[3], Takahiro Yamane[1,2], Keisuke Yatsu[4,5], Eiichi Okada[6], Nobuhisa Mizuki[1,2]

[1]Department of Ophthalmology and Visual Science, Yokohama City University Graduate School of Medicine, Yokohama, Kanagawa 236-0004, Japan
[2]Department of Advanced Medicine for Ocular Diseases, Yokohama City University Graduate School of Medicine, Yokohama, Kanagawa 236-0004, Japan
[3]Department of Public Health, Kyorin University School of Medicine, Mitaka, Tokyo 181-8611, Japan
[4]Department of Medical Science and Cardiorenal Medicine, Yokohama City University School of Medicine, Yokohama, Kanagawa 236-0004, Japan
[5]Minna no Naika Clinic Ningyocho-Suitengu, Chuo-ku, Tokyo 103-0013, Japan
[6]Okada Eye Clinic, Yokohama, Kanagawa 234-0054, Japan
[*]Corresponding author: akmeguro@yokohama-cu.ac.jp

CONTENTS

<u>Supplementary Tables</u>

Supplementary Table S1. Number of eyes assessed in this study.

Supplementary Table S2. Change in spherical equivalent (SE) refractive error over 5 years at each age at baseline.

Supplementary Table S3. Summary of changes in spherical equivalent (SE) refractive error reported by previous longitudinal studies.

<u>Supplementary References</u>

Supplementary Table S1. Number of eyes assessed in this study

SE at age at baseline, diopters	Number (%)* of eyes													Age-standardized prevalence of each SE group at all ages, %
	Age group at baseline, years													
	3–9	10–14	15–19	20–24	25–29	30–34	35–39	40–44	45–49	50–54	55–59	60–91	All ages	
Male														
-8.75 or less	0 (0.0)	23 (0.2)	353 (0.9)	826 (1.9)	1,056 (2.5)	1,069 (2.9)	832 (3.5)	692 (4.7)	516 (6.5)	359 (8.5)	169 (6.5)	44 (2.0)	5,939 (2.6)	3.09
-6.75 to -8.50	1 (0.2)	262 (2.7)	2,154 (5.4)	3,467 (7.9)	3,758 (9.0)	3,518 (9.6)	2,463 (10.3)	1,688 (11.5)	1,058 (13.3)	512 (12.1)	285 (11.0)	91 (4.1)	19,257 (8.4)	7.78
-4.75 to -6.50	11 (2.2)	1,343 (13.8)	8,213 (20.6)	10,778 (24.5)	10,934 (26.3)	9,384 (25.6)	6,024 (25.2)	3,733 (25.4)	2,048 (25.8)	1,064 (25.2)	547 (21.1)	318 (14.4)	54,397 (23.9)	20.01
-2.75 to -4.50	84 (17.1)	3,980 (40.9)	16,894 (42.3)	18,303 (41.5)	16,334 (39.3)	14,520 (39.5)	9,026 (37.7)	5,108 (34.7)	2,556 (32.2)	1,285 (30.5)	770 (29.7)	426 (19.2)	89,286 (39.1)	33.87
-0.75 to -2.50	278 (56.6)	3,825 (39.3)	11,901 (29.8)	10,436 (23.7)	9,215 (22.2)	7,950 (21.6)	5,336 (22.3)	3,239 (22.0)	1,504 (18.9)	739 (17.5)	514 (19.8)	458 (20.7)	55,395 (24.3)	29.13
+0.50 to -0.50	48 (9.8)	224 (2.3)	372 (0.9)	200 (0.5)	200 (0.5)	238 (0.6)	201 (0.8)	174 (1.2)	181 (2.3)	177 (4.2)	204 (7.9)	384 (17.3)	2,603 (1.1)	3.19
+2.50 to +0.75	34 (6.9)	34 (0.3)	17 (0.04)	3 (0.007)	9 (0.02)	18 (0.05)	19 (0.1)	23 (0.2)	28 (0.4)	56 (1.3)	96 (3.7)	434 (19.6)	771 (0.3)	1.55
+2.75 or more	35 (7.1)	38 (0.4)	28 (0.1)	49 (0.1)	38 (0.1)	24 (0.1)	23 (0.1)	46 (0.3)	53 (0.7)	22 (0.5)	10 (0.4)	60 (2.7)	426 (0.2)	1.37
All SE	491 (100.0)	9,729 (100.0)	39,932 (100.0)	44,062 (100.0)	41,544 (100.0)	36,721 (100.0)	23,924 (100.0)	14,703 (100.0)	7,944 (100.0)	4,214 (100.0)	2,595 (100.0)	2,215 (100.0)	228,074 (100.0)	
Females														
-8.75 or less	0 (0.0)	47 (0.3)	405 (0.7)	1,071 (1.6)	1,315 (2.2)	1,183 (2.4)	1,121 (3.1)	885 (3.0)	664 (3.2)	490 (3.6)	302 (4.0)	161 (4.3)	7,644 (2.1)	1.99
-6.75 to -8.50	1 (0.1)	404 (2.5)	2,957 (4.8)	5,005 (7.3)	4,825 (8.2)	3,923 (8.1)	3,159 (8.7)	2,478 (8.3)	1,850 (8.8)	1,226 (9.1)	664 (8.8)	226 (6.0)	26,718 (7.3)	6.28
-4.75 to -6.50	23 (3.3)	2,445 (15.1)	12,842 (21.0)	16,654 (24.3)	14,212 (24.2)	11,559 (23.9)	8,419 (23.3)	7,103 (23.8)	5,007 (23.9)	3,482 (25.9)	1,981 (26.4)	674 (17.9)	84,401 (23.1)	19.90
-2.75 to -4.50	133 (18.9)	6,137 (38.0)	25,883 (42.2)	27,252 (39.8)	23,103 (39.4)	19,117 (39.6)	14,157 (39.1)	11,863 (39.8)	8,372 (40.0)	5,151 (38.3)	2,630 (35.0)	991 (26.3)	144,789 (39.6)	35.95
-0.75 to -2.50	412 (58.4)	6,481 (40.1)	18,388 (30.0)	17,861 (26.1)	14,733 (25.1)	12,127 (25.1)	9,047 (25.0)	7,063 (23.7)	4,343 (20.7)	2,107 (15.7)	1,021 (13.6)	460 (12.2)	94,043 (25.8)	30.29
+0.50 to -0.50	73 (10.4)	570 (3.5)	690 (1.1)	524 (0.8)	359 (0.6)	285 (0.6)	191 (0.5)	230 (0.8)	323 (1.5)	311 (2.3)	214 (2.8)	355 (9.4)	4,125 (1.1)	2.84
+2.50 to +0.75	34 (4.8)	41 (0.3)	41 (0.1)	43 (0.1)	17 (0.03)	35 (0.1)	40 (0.1)	85 (0.3)	253 (1.2)	491 (3.7)	464 (6.2)	605 (16.1)	2,149 (0.6)	1.63
+2.75 or more	29 (4.1)	25 (0.2)	66 (0.1)	76 (0.1)	52 (0.1)	69 (0.1)	48 (0.1)	126 (0.4)	137 (0.7)	174 (1.3)	239 (3.2)	289 (7.7)	1,330 (0.4)	1.13
All SE	705 (100.0)	16,150 (100.0)	61,272 (100.0)	68,486 (100.0)	58,616 (100.0)	48,298 (100.0)	36,182 (100.0)	29,833 (100.0)	20,949 (100.0)	13,432 (100.0)	7,515 (100.0)	3,761 (100.0)	365,199 (100.0)	

SE, spherical equivalent; CI, confidence interval.
*Percentage of each SE group within each age group.

Supplementary Table S2. Change in spherical equivalent (SE) refractive error over 5 years at each age at baseline

	5-year mean change in spherical equivalent				
	Male		Female		Male vs. female
Age at baseline, years	N	Mean change (standard error)†, diopters	N	Mean change (standard error)†, diopters	$P^{\#}$
3	0	-	2	-0.750 (0.395)	
4	12	-0.479 (0.162)*	4	0.000 (0.279)	
5	12	-0.979 (0.162)*	20	-1.825 (0.125)*	
6	32	-2.180 (0.099)*	34	-3.081 (0.096)*	
7	68	-2.342 (0.068)*	66	-3.008 (0.069)*	<0.05
8	138	-2.654 (0.048)*	213	-3.110 (0.038)*	<0.01
9	229	-2.599 (0.037)*	366	-2.790 (0.029)*	
10	441	-2.416 (0.027)*	648	-2.502 (0.022)*	
11	777	-2.169 (0.020)*	1,183	-2.148 (0.016)*	
12	1,433	-1.672 (0.015)*	2,335	-1.712 (0.012)*	
13	2,956	-1.417 (0.010)*	5,005	-1.444 (0.008)*	
14	4,122	-1.224 (0.009)*	6,979	-1.245 (0.007)*	
15	5,707	-0.986 (0.007)*	9,013	-0.956 (0.006)*	<0.05
16	7,777	-0.805 (0.006)*	11,500	-0.758 (0.005)*	<0.001
17	8,400	-0.702 (0.006)*	12,562	-0.629 (0.005)*	<0.001
18	8,850	-0.567 (0.006)*	13,635	-0.486 (0.005)*	<0.001
19	9,198	-0.445 (0.006)*	14,562	-0.396 (0.005)*	<0.001
20	9,179	-0.391 (0.006)*	14,374	-0.363 (0.005)*	<0.001
21	9,032	-0.371 (0.006)*	14,059	-0.344 (0.005)*	<0.001
22	8,664	-0.364 (0.006)*	13,676	-0.328 (0.005)*	<0.001
23	8,529	-0.346 (0.006)*	13,452	-0.309 (0.005)*	<0.001
24	8,658	-0.314 (0.006)*	12,925	-0.282 (0.005)*	<0.001
25	8,483	-0.277 (0.006)*	12,492	-0.250 (0.005)*	<0.001
26	8,456	-0.262 (0.006)*	12,229	-0.220 (0.005)*	<0.001
27	8,219	-0.217 (0.006)*	11,812	-0.183 (0.005)*	<0.001
28	8,248	-0.213 (0.006)	11,214	-0.168 (0.005)	<0.001
29	8,138	-0.182 (0.006)	10,869	-0.135 (0.005)	<0.001
30	7,998	-0.165 (0.006)	10,512	-0.114 (0.005)	<0.001
31	7,851	-0.137 (0.006)	10,070	-0.103 (0.006)	<0.001
32	7,565	-0.124 (0.006)	9,806	-0.081 (0.006)	<0.001
33	6,926	-0.121 (0.007)	9,206	-0.070 (0.006)	<0.001
34	6,381	-0.094 (0.007)	8,704	-0.050 (0.006)	<0.001
35	5,712	-0.089 (0.007)	8,161	-0.050 (0.006)	<0.001
36	5,224	-0.090 (0.008)	7,439	-0.056 (0.006)	<0.001
37	4,669	-0.089 (0.008)	7,128	-0.057 (0.007)	<0.001
38	4,377	-0.097 (0.009)	6,864	-0.058 (0.007)	<0.001
39	3,942	-0.101 (0.009)	6,590	-0.059 (0.007)	<0.001
40	3,583	-0.087 (0.009)	6,561	-0.069 (0.007)	
41	3,132	-0.098 (0.010)	6,127	-0.076 (0.007)	<0.001
42	2,989	-0.098 (0.010)	6,105	-0.081 (0.007)	
43	2,640	-0.101 (0.011)	5,735	-0.084 (0.007)	
44	2,359	-0.101 (0.012)	5,305	-0.099 (0.008)	
45	2,001	-0.089 (0.013)	4,906	-0.084 (0.008)	
46	1,773	-0.043 (0.013)	4,516	-0.092 (0.008)	<0.001
47	1,579	-0.042 (0.014)	4,234	-0.070 (0.009)	
48	1,373	-0.012 (0.015)	3,810	-0.053 (0.009)	<0.05
49	1,218	-0.029 (0.016)	3,483	-0.014 (0.009)	
50	1,051	-0.014 (0.017)	3,215	-0.008 (0.010)	
51	892	+0.003 (0.019)	2,940	+0.021 (0.010)	
52	825	+0.047 (0.020)	2,553	+0.038 (0.011)	
53	736	+0.055 (0.021	2,487	+0.055 (0.011)	
54	710	+0.105 (0.021)	2,237	+0.064 (0.012)	
55	614	+0.180 (0.023)	2,087	+0.064 (0.012)	<0.001
56	623	+0.160 (0.023)	1,755	+0.081 (0.013)	<0.001

Supplementary Table S2. (Cont.)

Age at baseline, years	5-year mean change in spherical equivalent				Male vs. female
	Male		Female		
	N	Mean change (standard error)†, diopters	N	Mean change (standard error)†, diopters	$P^{\#}$
57	517	+0.185 (0.025)	1,460	+0.099 (0.015)	<0.001
58	443	+0.157 (0.027)	1,248	+0.083 (0.016)	<0.01
59	398	+0.195 (0.028)	965	+0.115 (0.018)	<0.05
60	345	+0.196 (0.030)	705	+0.123 (0.021)	<0.01
61	185	+0.246 (0.041)	526	+0.057 (0.022)	<0.001
62	206	+0.358 (0.039)	450	+0.163 (0.026)	<0.001
63	201	+0.228 (0.040)	417	+0.189 (0.026)	
64	170	+0.394 (0.040)	340	+0.101 (0.030)	<0.001
65	170	+0.231 (0.043)	254	+0.101 (0.033)	<0.05
66	173	+0.363 (0.043)	218	+0.173 (0.036)	<0.001
67	127	+0.145 (0.049)	204	+0.202 (0.039)	
68	82	+0.322 (0.059)	106	+0.099 (0.051)	<0.01
69	83	+0.241 (0.062)	130	+0.087 (0.048)	<0.05
70	65	+0.127 (0.070)	81	+0.103 (0.060)	
71	94	+0.136 (0.058)	64	+0.083 (0.066)	
72	70	+0.186 (0.067)	29	-0.017 (0.097)	<0.05
73	44	+0.142 (0.079)	38	+0.184 (0.085)	
74	35	+0.183 (0.093)	34	-0.108 (0.081)	<0.05
75	23	-0.139 (0.121)	27	-0.187 (0.103)	
76	39	+0.090 (0.090)	25	+0.200 (0.095)	
77	22	+0.315 (0.088)	15	+0.083 (0.131)	
78	14	+0.232 (0.150)	13	-0.192 (0.122)	
79	21	+0.222 (0.119)	14	-0.500 (0.125)	<0.01
80	16	+0.219 (0.131)	3	+0.100 (0.524)	
81	11	0.000 (0.170)	13	+0.135 (0.155)	
82	5	-0.100 (0.252)	12	+0.042 (0.161)	
83	1	0.000 (0.000)	10	+0.800 (0.176)	
84	6	0.000 (0.230)	11	+0.182 (0.158)	
85	4	+0.500 (0.281)	6	+0.250 (0.228)	
86	2	0.000 (0.398)	9	+0.083 (0.186)	
87	0	-	2	+0.250 (0.395)	
88	1	0.000 (0.000)	2	0.000 (0.395)	
89	0	-	0	-	
90	0	-	1	+0.500 (0.000)	
91	0	-	2	+0.250 (0.395)	

†5-year mean change (standard error) in SE adjusted by analysis of covariance with SE at age at baseline as an independent variable.

*5-year mean SE changes significant at $P < 0.05$ after Bonferroni correction based on the number of ages investigated for each sex.

#P values that showed a significant sex difference ($P < 0.05$) in 5-year mean SE change are listed.

Supplemental Table S3. Summary of changes in spherical equivalent (SE) refractive error reported by previous longitudinal studies

Study [Reference]	Population	Number of eyes followed up	Age at baseline (years)	Follow-up period (years)	Key findings/conclusions	Notes
Mäntyjärvi et al., 1985 [1]	Finnish	1,118	7–15	5–8	<u>Mean annual SE change during study period</u><br>• Baseline SE state<br>Myopia: -0.55 ± 0.27 D<br>Hyperopia: -0.12 ± 0.14 D<br>• Largest annual myopic SE change (-0.93 ± 0.65 D) in myopic children age 8-9 y<br>• Faster myopic progression in children with myopia vs with hyperopia	
Grosvenor et al., 1993 [2]	New Zealander	53	Young adults	3	<u>Mean SE change during 3-year period</u><br>• Baseline SE state<br>Youth-onset myopia: -0.26 ± 0.52 D<br>Early adult-onset myopia: -0.18 ± 0.40 D<br>Emmetropia: -0.15 ± 0.87 D<br>• Youth onset associated with significantly more myopia than early adult onset	<u>Myopia/emmetropia definition</u><br>• Youth onset myopia: SE ≤ -0.50 D, age < 16 y<br>• Early adult onset myopia: SE ≤ -0.50 D, age ≥ 16 y<br>• Emmetropia: -0.50 D < SE ≤ +1.00 D
McBrien et al., 1997 [3]	British	332	21–55	2	<u>Mean SE change during 2-year period</u><br>• The higher the myopic level at study start, the faster the myopic shift	
Shih et al., 2001 [4]	Taiwanese	61	6–13	1.5	<u>Mean SE change during 1.5-year period</u><br>• Baseline SE state<br>Myopia: -1.40 ± 0.09 D	<u>Participants information</u><br>• Mean SE of-3.20 ± 0.14 D at baseline in myopic children
Zhao et al., 2002 [5]	Chinese	9,324	5–13	28.5 months	<u>Mean SE change during 28.5-month period</u><br>• Baseline SE state<br>No myopia: -0.36 D<br>Myopia: -0.84 D<br>• Sex<br>Male: -0.32 ± 0.65 D<br>Female: -0.53 ± 0.70 D<br>• Female sex associated with faster myopic progression	<u>Myopia definition</u><br>• No myopia: SE > -0.50 D<br>• Myopia: SE ≤ -0.50 D<br><br><u>Additional finding</u><br>• Older age, higher myopic or hyperopic refractive error at baseline associated with faster myopic
Lee et al., 2002 [6]	US cohort:<br>predominantly white population	2,362	43–84	10	<u>Mean SE change during 10-year period</u><br>• Age group<br>43–59 y: +0.48 D<br>60–69 y: +0.03 D<br>70 y or older: -0.19 D<br>• Younger people shifted more hyperopic, whereas older people shifted more myopic	
Guzowski et al., 2003 [7]	Australian:<br>predominantly white population	1,850	≥49	5	<u>Mean SE change during 5-year period</u><br>• Age group<br>49–54 y: +0.41 D<br>55–64 y: +0.30 D<br>65–74 y: +0.04 D<br>75 y or older: -0.22 D<br>• Younger people shifted more hyperopic, whereas older people shifted more myopic	<u>Additional finding</u><br>• Myopic shift in older population associated with development of nuclear cataract
Fan et al., 2004 [8]	Chinese (Hong-Kong)	7,560	5–16	1	<u>Mean SE change during 1-year period</u><br>• Baseline SE state<br>Emmetropia: -0.29 D<br>Low myopia: -0.63 D<br>Moderate myopia: -0.64 D<br>High myopia: -0.71 D<br>• Higher degree of myopia at baseline associated with steeper annual myopic progression	<u>Myopia/emmetropia definition</u><br>• Emmetropia: -0.50 D < SE < 2.00 D<br>• Low myopia: -3.00 D < SE ≤ -0.50 D<br>• Moderate myopia: -6.00 D < SE ≤ -3.00 D<br>• High myopia: SE ≤ -6.00 D
Tan et al., 2005 [9]	Singaporean cohort:<br>Chinese, 99.4%<br>Other, 0.6%	706	6–12	1	<u>Mean SE change during 1-year period</u><br>• Myopic children: -0.84 D	<u>Participants information</u><br>• Enrolled myopic children with SE of -0.75 D to -4.00 D at baseline
Saw et al., 2005 [10]	Singaporean multi-ethnic cohort:<br>Chinese (majority population), Malaysian, and Indian	842	7–9	3	<u>Mean SE change during 3-year period in entire (myopic and non-myopic) children</u><br>• Baseline SE state<br>No myopia: -1.00 D<br>Low myopia: -2.07 D<br>Higher myopia: -2.33 D<br>High myopia: -1.56 D<br><u>Mean SE change during 3-year period in myopic children</u><br>• Age group<br>7 y: -2.40 D<br>8 y: -1.97 D<br>9 y: -1.71 D<br>• Sex<br>Male: -1.88 D<br>Female: -2.38 D<br>• Younger baseline age, female sex associated with faster myopic progression in myopic children	<u>Myopia definition</u><br>• No myopia: SE > -0.50 D<br>• Low myopia: -3.00 D < SE ≤ -0.50 D<br>• Higher myopia: -6.00 D < SE ≤ -3.00 D<br>• High myopia: SE ≤ -6.00 D<br><br><u>Additional finding</u><br>• Faster myopia progression in children of Chinese ethnicity vs children of non-Chinese (Malaysian and Indian) ethnicity
Hyman, et al., 2005 [11]	US multi-ethnic cohort:<br>White, 47.4%<br>Black, 26.1%<br>Hispanic, 15.0%<br>Asian, 6.0%<br>Mixed, 5.6%	468	6–11	3	<u>Mean SE change during 3-year period</u><br>• Age group<br>6-7 y: -2.19 ± 0.21 D<br>8 y: -1.78 ± 0.14 D<br>9 y: -1.45 ± 0.09 D<br>10 y: -1.23 ± 0.08 D<br>11 y: -1.04 ± 0.10 D<br>• Sex<br>Male: -1.33 ± 0.07 D<br>Female: -1.49 ± 0.07 D<br>• Younger baseline age, female sex associated with faster myopic progression	<u>Data information</u><br>• Data for mean SE change in children who wore single vision lenses<br><br><u>Additional finding</u><br>• Faster myopia progression in white children or children of Hispanic, Asian, or mixed ethnicity vs Black children
Wu et al., 2005 [12]	Barbadian:<br>predominantly of African descent	2,128	≥40	9	<u>Mean SE change during 9-year period</u><br>• Age group<br>40–49 y: +0.47 ± 0.75 D<br>50–59 y: -0.11 ± 1.08 D<br>60–69 y: -0.90 ± 1.41 D<br>70 y or older: -0.93 ± 1.03 D<br>• Younger people shifted more hyperopic, whereas older people shifted more myopic	
Siatkowski et al., 2008 [13]	US multi-ethnic cohort:<br>White, 74.2%<br>Black, 6.5%<br>Asian, 3.2%<br>Hispanic, 12.9%<br>Other, 3.2%	62	8–12	2	<u>Mean SE change during 1-year period</u><br>• Baseline SE state<br>Myopia: -0.56 ± 0.51 D<br><u>Mean SE change during 2-year period</u><br>• Baseline SE state<br>Myopia: -0.99 ± 0.68 D	<u>Participants information</u><br>• Enrolled myopic children with SE of -0.75 D to -4.00 D at baseline

Supplemental Table S3. (Cont.)

Study [Reference]	Population	Number of eyes followed up	Age at baseline (years)	Follow-up period (years)	Key findings/conclusions	Notes
Hasebe et al., 2008 [14]	Japanese	80	6–12	1.5	<u>Mean SE change during 1.5-year period</u><br>• Baseline SE state<br>Less myopia: -1.14 D<br>More myopia: -1.30 D<br>• Age group<br>Younger children (age <10 y): -1.36 D<br>Older children (age ≥ 10 y): -1.03 D<br>• Greater myopia progression observed in more myopic eyes and younger ages	<u>Myopia definition</u><br>• Less myopia: SE > -3.3 D<br>• More myopia: SE ≤ -3.3 D
Fotedar et al., 2008 [15]	Australian:<br>predominantly white population	1,336	≥49	10	<u>Mean SE change during 10-year period</u><br>• Age group<br>49–54 y: +0.40 D<br>55–64 y: +0.33 D<br>65–74 y: -0.02 D<br>75 y or older: -0.65 D<br>• Younger people shifted more hyperopic, whereas older people shifted more myopic	<u>Additional finding</u><br>• Myopic shift in older population associated with development of nuclear cataract
Yang et al., 2009 [16]	Chinese	150	7–13	2	<u>Mean SE change during 2-year period</u><br>• Baseline SE state<br>Myopia: -1.50 ± 0.67 D<br>• Sex<br>Male: -1.39 ± 0.66 D<br>Female: -1.67 ± 0.79 D<br>• Faster myopic progression in females vs males	<u>Participants information</u><br>• Enrolled myopic children with SE of -0.50 D to -3.00 D at baseline<br><br><u>Data information</u><br>• Data for mean SE change in children who wore single vision lenses
Tong et al., 2009 [17]	Singaporean multi-ethnic cohort:<br>Chinese, 93.0%<br>Indian, 5.0%<br>Other, 2.0%	200	6–12	1 and 3	<u>Mean SE change during 1-year period</u><br>• Baseline SE state<br>Myopia: -0.38 ± 0.39 D<br><u>Mean SE change during 3-year period</u><br>• Baseline SE state<br>Myopia: -0.52 ± 0.30 D/year	<u>Participants information</u><br>• Enrolled myopic children with SE of -1.00 D to -6.00 D and astigmatism of -1.50 D or less at baseline
Xiang et al., 2012 [18]	Chinese	607	7–15	4	<u>Mean annual SE change</u><br>• During 4-year period before myopia onset: -0.25 D<br>• During 2-year period before myopia onset: -0.40 D<br>• During 1-year period before myopia onset: -0.92 D<br>• During 1-year period after myopia onset: -0.71 D<br>• During 2-year period after myopia onset: -0.65 D<br>• During 3-year period after myopia onset: -0.34 D<br>• Before myopia onset, axial elongation and myopic change accelerated; after myopic refraction established, axial elongation and myopic progression decreased<br>• Larger annual SE changes in younger (7–11 years) vs older children (12–15 years)	
Lv et al., 2013 [19]	Chinese	2,053	Mean age: 18.27	2	<u>Mean SE change during 2-year period</u><br>• Baseline SE state<br>Hyperopia: -0.22 ± 0.23 D<br>Emmetropia: -0.18 ± 0.15 D<br>Low myopia: -0.28 ± 0.31 D<br>Moderate myopia: -0.42 ± 0.43 D<br>High myopia: -0.52 ± 0.37 D<br>• Sex<br>Male: -0.29 ± 0.37 D<br>Female: -0.35 ± 0.31 D<br>• The higher the myopic level at study start, the faster the myopic shift<br>• Significantly faster myopic progression in females vs males	<u>Participants information</u><br>• All new university students<br><br><u>Myopia/emmetropia/hyperopia definition</u><br>• Hyperopia: SE ≥ +0.50 D<br>• Emmetropia:-0.50 D < SE < +0.50 D<br>• Low myopia: -3.00 D < SE ≤ -0.50 D<br>• Moderate myopia: -6.00 D < SE ≤ -3.00 D<br>• High myopia: SE ≤ -6.00 D
French et al., 2013 [20]	Australian multi-ethnic cohort:<br><u>Younger cohort</u><br>European white, 67.5%<br>East Asian, 15.9%<br>Other, 16.7%<br><u>Older cohort</u><br>European white, 57.2%<br>East Asian, 19.4%<br>Other, 23.4%	2,059	Mean age: younger cohort, 6.7; older cohort, 12.7	5-6	<u>Mean annual SE change during study period</u><br>• Younger cohort<br>All children: -0.16 D<br>Sex–<br>Male: -0.15 D<br>Female: -0.17 D<br>Baseline SE state–<br>Significant hyperopia: -0.19 D<br>Emmetropia: -0.15 D<br>Myopia: -0.41 D<br>• Older cohort<br>All children: -0.15 D<br>Sex–<br>Male: -0.13 D<br>Female: -0.15 D<br>Baseline SE state–<br>Significant hyperopia: -0.26 D<br>Emmetropia: -0.11 D<br>Myopia: -0.31 D<br>• Steeper myopic progression in children with ametropia (myopia or hyperopia) at baseline vs children with emmetropia<br>• Slightly faster myopic progression in females vs males	<u>Myopia/emmetropia/hyperopia definition</u><br>• Significant hyperopia: SE ≥ +2.00 D<br>• Mild hyperopia: +0.50 ≤ SE < +2.00 D<br>• Emmetropia: -0.50 D < SE < +0.50 D<br>• Myopia: SE ≤ -0.50 D<br><br><u>Additional finding</u><br>• For children with myopia at study start, no difference in myopia progression between children of European white and East Asian ethnicity
Hashemi et al., 2016 [21]	Iranian	3,960	40–64	5	<u>Mean SE change during 5-year period</u><br>• Age group<br>40–44 y: +0.09 D<br>45–49 y: +0.29 D<br>50–54 y: +0.34 D<br>55–59 y: +0.25 D<br>60–64 y: +0.10 D<br>• Sex<br>Male: +0.19 D<br>Female: +0.27 D<br>• Hyperopic shift greater in females than males	<u>Additional finding</u><br>• Myopic shift in older population associated with development of nuclear cataract

Supplemental Table S3. (Cont.)

Study [Reference]	Population	Number of eyes followed up	Age at baseline (years)	Follow-up period (years)	Key findings/conclusions	Notes
Zhou et al., 2016 [22]	Chinese	1,858	6–15	5	<u>Mean SE change during 5-year period</u><br>• Entire study cohort: -2.21 ± 1.87 D<br>• Sex<br>Male: -1.99 ± 1.88 D<br>Female: -2.41 ± 1.83 D<br>• Younger baseline age, female sex associated with faster myopic progression<br>• Higher myopic and hyperopic SE at baseline also associated with faster myopic progression	<u>Participants information</u><br>• Mean SE change in baseline myopic eyes of -3.56 D versus -1.32 D in all other eyes
Han et al., 2017 [23]	Chinese	1,300	≥35	6	<u>Mean SE change during 6-year period</u><br>• Age group<br>35–44 y: +0.24 D<br>45–54 y: +0.51 D<br>55–64 y: +0.26 D<br>65 y or older: -0.05 D<br>• Sex<br>Male: +0.34 D<br>Female: +0.29 D<br>• Baseline SE state<br>Hyperopia: +0.38 D<br>Emmetropia: +0.41 D<br>Mild myopia: +0.18 D<br>Moderate to high myopia: +0.01 D<br>• Hyperopic shift in SE change for elderly age <65 years and myopic shift thereafter<br>• Baseline SE state related to a hyperopic shift in SE	<u>Myopia/emmetropia/hyperopia definition</u><br>• Hyperopia: SE > +0.50 D<br>• Emmetropia:-0.50 D ≤ SE ≤ +0.50 D<br>• Mild myopia: -3.00 D ≤ SE < -0.50 D<br>• Moderate to high myopia: SE < -3.00 D<br><br><u>Additional finding</u><br>• Myopic shift in older population associated with development of nuclear cataract
Li et al., 2018 [24]	Chinese	3,970	≥30	5	<u>Mean SE change during 5-year period</u><br>• Age group<br>30–39 y: -0.21 D<br>40–49 y: +0.14 D<br>50–59 y: +0.40 D<br>60–69 y: +0.08 D<br>70 y or older: -0.34 D<br>• Sex<br>Male: +0.15 D<br>Female: +0.18 D<br>• Baseline SE state<br>Hyperopia: +0.27 D<br>Emmetropia: +0.20 D<br>Myopia: -0.05 D<br>• Hyperopic shift with aging, maximum during ages 50–59 y, then decrease and switch to a myopic shift again after 70 y<br>• Baseline SE state positively associated with SE change	<u>Myopia/emmetropia/hyperopia definition</u><br>• Hyperopia: SE > +0.50 D<br>• Emmetropia: -0.50 D ≤ SE ≤ +0.50 D<br>• Myopia: SE < -0.50 D
Wong et al., 2020 [25]	Mult-ethnic	3,355	<17	Mean follow-up period: 2	<u>Mean annual SE change during study period in children who experienced myopia progression during a follow-up</u><br>• Sex<br>Male: -0.38 D<br>Female: -0.42 D<br>• Baseline SE state<br>Mild myopia: -0.37 D<br>Moderate myopia: -0.54 D<br>• Age of puberty (average age of onset of puberty, 12 years in boys and 11 years in girls)<br>Before puberty: -0.41 D<br>After puberty: -0.35 D<br>• Slightly faster myopic progression in females vs males<br>• Faster myopic progression in children with moderate myopia at baseline vs children with mild myopia<br>• Myopic progression faster before vs after average age of onset of puberty	<u>Participants information</u><br>• 3,355 children with mild/moderate myopia at study start<br>• Myopia progression in 2,095 of 3,355 children during follow-up<br><br><u>Myopia/emmetropia definition</u><br>• Emmetropia:-0.50 D < SE < +0.50 D<br>• Mild myopia: -3.00 D < SE ≤ -0.50 D<br>• Moderate myopia: -6.00 D < SE ≤ -3.00 D<br>• High myopia: -12.00 D < SE ≤ -6.00 D
Verkicharla et al., 2020 [26]	Indian	6,984	1–30	1	<u>Mean SE change during 1-year period</u><br>• Age group<br>0–5 y: -0.49 ± 0.86 D<br>6–10 y: -0.51 ± 0.79 D<br>11–15 y: -0.46 ± 0.68 D<br>16–20 y: -0.23 ± 0.56 D<br>21–25 y: -0.11 ± 0.51 D<br>26–30 y: -0.07 ± 0.54 D<br>• Sex<br>Male: -0.31 ± 0.66 D<br>Female: -0.36 ± 0.70 D<br>• Baseline SE state<br>Mild myopia: -0.29 ± 0.60 D<br>Moderate myopia: -0.36 ± 0.69 D<br>High myopia: -0.40 ± 0.81 D<br>Severe myopia: -0.67 ± 1.10 D<br>• Identified largest annual change in SE in children ages 6–10 years, followed by those ages 0–5 and 11–15 years<br>• Greatest annual myopic progression seen with severe myopia, followed by high, moderate, and mild myopia, in both younger (0–15 y) and older (16–30 y) age groups	<u>Myopia/emmetropia definition</u><br>• Mild myopia: -3.00 D ≤ SE ≤ -0.50 D<br>• Moderate myopia: -6.00 D ≤ SE < -3.00 D<br>• High myopia: -9.00 D ≤ SE < -6.00 D<br>• Severe myopia: SE < 9.00 D<br><br><u>Additional finding</u><br>• Early onset of myopia associated with high myopia in adulthood
Jones-Jordan et al., 2021 [27]	US multi-ethnic cohort:<br>White, 52.7%<br>Hispanic, 26.8%<br>Black, 14.2%<br>Asian American, 5.5%<br>Native American, 0.8%	594	7–13	1	<u>Mean SE change in the first year following myopia onset</u><br>• Onset age<br>7 y: -0.58 D<br>8 y: -0.51 D<br>9 y: -0.44 D<br>10 y: -0.37 D<br>11 y: -0.30 D<br>12 y: -0.23 D<br>13 y: -0.16 D<br>• Younger age, female sex associated with faster myopic progression	<u>Myopia definition</u><br>• Myopia: SE ≤ -0.75 D<br><br><u>Additional finding</u><br>• Faster myopic progression in Asian American children vs Black, Hispanic, or Native American children

Supplementary References

1. Mäntyjärvi, M. I. Changes of refraction in schoolchildren. *Arch. Ophthalmol.* **103**, 790-792 (1985).
2. Grosvenor, T. & Scott, R. Three-year changes in refraction and its components in youth-onset and early adult-onset myopia. *Optom. Vis. Sci.* **70**, 677-683 (1993).
3. McBrien, N. A. & Adams, D. W. A longitudinal investigation of adult-onset and adult-progression of myopia in an occupational group. Refractive and biometric findings. *Invest. Ophthalmol. Vis. Sci.* **38**, 321-333 (1997).
4. Shih, Y. F. *et al*. An intervention trial on efficacy of atropine and multi-focal glasses in controlling myopic progression. *Acta Ophthalmol. Scand.* **79**, 233-236 (2001).
5. Zhao, J. *et al*. The progression of refractive error in school-age children: Shunyi district, China. *Am. J. Ophthalmol.* **134**, 735-743 (2002).
6. Lee, K. E., Klein, B. E., Klein, R. & Wong, T. Y. Changes in refraction over 10 years in an adult population: the Beaver Dam Eye study. *Invest. Ophthalmol. Vis. Sci.* **43**, 2566-2571 (2002).
7. Guzowski, M., Wang, J. J., Rochtchina, E., Rose, K. A. & Mitchell, P. Five-year refractive changes in an older population: the Blue Mountains Eye Study. *Ophthalmology.* **110**, 1364-1370 (2003).
8. Fan, D. S. *et al*. Prevalence, incidence, and progression of myopia of school children in Hong Kong. *Invest. Ophthalmol. Vis. Sci.* **45**, 1071-1075 (2004).
9. Tan, D. T. *et al*. One-year multicenter, double-masked, placebo-controlled, parallel safety and efficacy study of 2% pirenzepine ophthalmic gel in children with myopia. *Ophthalmology.* **112**, 84-91 (2005).
10. Saw, S. M. *et al*. Incidence and progression of myopia in Singaporean school children. *Invest. Ophthalmol. Vis. Sci.* **46**, 51-57 (2005).
11. Hyman, L. *et al*. Relationship of age, sex, and ethnicity with myopia progression and axial elongation in the correction of myopia evaluation trial. *Arch. Ophthalmol.* **123**, 977-987 (2005).
12. Wu, S. Y. *et al*. Nine-year refractive changes in the Barbados Eye Studies. *Invest. Ophthalmol. Vis. Sci.* **46**, 4032-4039 (2005).
13. Siatkowski, R. M. *et al*. Two-year multicenter, randomized, double-masked, placebo-controlled, parallel safety and efficacy study of 2% pirenzepine ophthalmic gel in children with myopia. *J. AAPOS.* **12**, 332-339 (2008).
14. Hasebe, S. *et al.* Effect of progressive addition lenses on myopia progression in Japanese children: a prospective, randomized, double-masked, crossover trial. *Invest. Ophthalmol. Vis. Sci.* **49**, 2781-2789 (2008).
15. Fotedar, R., Mitchell, P., Burlutsky, G. & Wang, J. J. Relationship of 10-year change in refraction to nuclear cataract and axial length findings from an older population. *Ophthalmology*. **115**, 1273-1278, 1278.e1271 (2008).

16. Yang, Z. *et al*. The effectiveness of progressive addition lenses on the progression of myopia in Chinese children. *Ophthalmic Physiol. Opt.* **29**, 41-48 (2009).

17. Tong, L. *et al*. Atropine for the treatment of childhood myopia: effect on myopia progression after cessation of atropine. *Ophthalmology.* **116**, 572-579 (2009).

18. Xiang, F., He, M. & Morgan, I. G. Annual changes in refractive errors and ocular components before and after the onset of myopia in Chinese children. *Ophthalmology.* **119**, 1478-1484 (2012).

19. Lv, L. & Zhang, Z. Pattern of myopia progression in Chinese medical students: a two-year follow-up study. *Graefes Arch. Clin. Exp. Ophthalmol.* **251**, 163-168 (2013).

20. French, A. N., Morgan, I. G., Burlutsky, G., Mitchell, P. & Rose, K. A. Prevalence and 5- to 6-year incidence and progression of myopia and hyperopia in Australian schoolchildren. *Ophthalmology.* **120**, 1482-1491 (2013).

21. Hashemi, H., Khabazkhoob, M., Iribarren, R., Emamian, M. H. & Fotouhi, A. Five-year change in refraction and its ocular components in the 40- to 64-year-old population of the Shahroud eye cohort study. *Clin. Exp. Ophthalmol.* **44**, 669-677 (2016).

22. Zhou, W. J. *et al*. Five-year progression of refractive errors and incidence of myopia in school-aged children in Western China. *J. Epidemiol.* **26**, 386-395 (2016).

23. Han, X., Guo, X., Lee, P. Y., Morgan, I. G. & He, M. Six-year changes in refraction and related ocular biometric factors in an adult Chinese population. *PLoS One.* **12**, e0183364 (2017).

24. Li, S. M. *et al.* Five-year refractive changes in a rural Chinese adult population and its related factors: the Handan Eye Study. *Clin. Exp. Ophthalmol*. **46**, 873-881 (2018).

25. Wong, K. & Dahlmann-Noor, A. Myopia and its progression in children in London, UK: a retrospective evaluation. *J. Optom.* **13**, 146-154 (2020).

26. Verkicharla, P. K., Kammari, P. & Das, A. V. Myopia progression varies with age and severity of myopia. *PLoS One.* **15**, e0241759 (2020).

27. Jones-Jordan, L. A. *et al.* Myopia Progression as a Function of Sex, Age, and Ethnicity. *Invest. Ophthalmol. Vis. Sci.* **62**, 36 (2021).
